# Malignancies in Celiac Disease—A Hidden Threat with Diagnostic Pitfalls

**DOI:** 10.3390/biomedicines13061507

**Published:** 2025-06-19

**Authors:** Aleksandra Kubas, Ewa Małecka-Wojciesko

**Affiliations:** Department of Digestive Tract Diseases, Medical University of Lodz, Kopcinskiego 22, 90-153 Lodz, Poland

**Keywords:** celiac disease, malignancy, refractory celiac disease, lymphoma, carcinoma, screening

## Abstract

Celiac disease (CeD) is an autoimmune disorder that is triggered by gluten ingestion in genetically predisposed individuals. Untreated or poorly controlled CeD leads to various disease complications, such as malnutrition, osteoporosis, autoimmune diseases, or refractory celiac disease (RCD). Accumulating recent research has highlighted the association between CeD and the development of malignancies, particularly enteropathy-associated T-cell lymphoma (EATL) and small bowel carcinoma (SBC), which are neoplasms with extremely poor prognoses. Genetic alterations in the JAK1–STAT3 pathway and the high prevalence of microsatellite instability may be the main drivers of CeD-associated lymphomagenesis and small bowel oncogenesis and therefore could be an attractive therapeutic target to block cancer transformation. However, to date, the risk factors and exact mechanisms underlying malignancy development in patients with CeD remain unclear, and prospective cohort studies that include molecular profiling are needed. Moreover, current guidelines on the management of CeD do not provide standardized protocols for cancer surveillance—particularly regarding screening intervals, risk stratification, and monitoring strategies for high-risk patients such as those with RCD. This paper reviews the existing knowledge on malignancies in CeD, highlights diagnostic challenges, and discusses future perspectives on the early detection, monitoring, and treatment of CeD-associated neoplasms.

## 1. Introduction

Celiac disease (CeD) is an autoimmune disorder that is triggered by gluten ingestion in genetically predisposed individuals. The global prevalence of CeD ranges from 0.7% based on histological examination to 1.4% based on serological markers with a clear female predominance (female-to-male ratio of approximately 2–2.5:1) [1]. The typical clinical manifestations of CeD are abdominal pain, diarrhea, and malnutrition, which lead to intestinal mucosal villous atrophy, crypt hyperplasia, and an increased intraepithelial lymphocyte (IEL) count [2]. Diagnosis relies on intestinal mucosal biopsy combined with serological and genetic tests [3]. Tissue samples are assessed according to the Marsh classification, which includes the presence of an increased number of IELs (>25 cells per 100 enterocytes), crypt hyperplasia, and villous atrophy. A normal histopathological image of the mucosa (Marsh 0) and an isolated increase in the number of IELs (Marsh 1) do not confirm the presence of CeD. Moreover, although genetic testing for CeD-compatible human leukocyte antigen (HLA) haplotypes is not mandatory in all cases, it might be useful among individuals who have already started a gluten-free diet (GFD) before evaluation or in situations in which serological and histological findings are inconsistent, as it has a high negative predictive value for CeD [3]. Owing to the variable clinical presentation of CeD and its potentially asymptomatic course, many patients remain undiagnosed for years [4].

Strict adherence to a GFD continues to be the gold standard for CeD treatment. Although most patients comply with dietary recommendations and achieve mucosal damage healing, adherence to a GFD is worse among certain groups of individuals, such as adolescents, those with lower awareness of the importance of diet, and patients who inadvertently consume small amounts of gluten [5,6,7]. This, along with older age and diagnostic delay, can lead to various disease complications, such as malnutrition, osteoporosis, autoimmune diseases, or refractory celiac disease (RCD) [8,9,10,11]. Importantly, data on the association between CeD and malignancy are accumulating [12,13,14,15,16]. To date, poor adherence to a GFD, non-responsive CeD (persistent or recurrent symptoms despite following GFD for 6 to 12 months), refractory celiac disease, *HLA-DQ2* homozygosity, and late (≥40 years) or recent (first year of follow-up) diagnosis of CeD have been proposed as risk factors for malignancy development in CeD [12,17]. Currently available guidelines for CeD treatment and monitoring do not provide clear recommendations for cancer screening and surveillance. Therefore, this study aimed to review the existing knowledge on malignancies in CeD and highlight the importance of developing prevention and monitoring strategies. To support this objective, we conducted a narrative review based on a comprehensive literature search. Scientific databases including PubMed, Google Scholar, and Scopus were used to identify relevant publications. Search terms such as “celiac disease”, “malignancy”, “lymphoma”, “intestinal cancer”, and “molecular” were used in various combinations. Preference was given to peer-reviewed studies, systematic reviews, and meta-analyses published within the past 10–15 years, although older works were also considered when appropriate.

## 2. Risk of Malignancies in Patients with CeD

The number of studies investigating the risk of malignancy in patients with CeD has increased. In a recent Swedish study including 47,241 patients with CeD, Lebwohl et al. reported an incidence rate of 6.5 cancer cases per 1000 person-years in patients with CeD, versus 5.7 per 1000 person-years in the general population. Interestingly, the risk was significantly elevated only in the first year following CeD diagnosis, with the highest risk observed in patients diagnosed after the age of 60 [12]. Other studies have confirmed the above-mentioned observations regarding the correlation between malignancy risk and the time and age at CeD diagnosis [17,18,19]. The decline in the malignancy risk 1 year after diagnosis may be attributable to mucosal healing after the implementation of a GFD or to increased surveillance among patients with CeD [12]. Various studies that have evaluated the risk of malignancy and cancer-related mortality in patients with CeD are presented in Table 1.

Neoplasms reported in CeD include malignant lymphomas and small-intestinal, oropharyngeal, esophageal, large intestinal, hepatobiliary, and pancreatic carcinomas [14,21,22,23,24]. Interestingly, two studies have reported that patients with CeD have a decreased risk of breast cancer [21,25]. The data on the risk of developing various malignancies in patients with CeD are summarized in Table 2.

The precise pathogenic mechanisms underlying the development of malignancies in patients with CeD remain unclear. However, chronic inflammation, continuous antigen stimulation, cytokine release, genome instability, and mutations are cancer hallmarks commonly shared by CeD [45,46,47]. Among the various factors contributing to cancer risk in CeD, RCD has gained particular attention as a potential premalignant condition. Although malignancies can develop in CeD independently of RCD, the persistent mucosal damage and immune activation seen in RCD—despite strict adherence to a GFD—suggest an environment highly conducive to neoplastic transformation. Notably, RCD, particularly type 2, is widely recognized as a key precursor to enteropathy-associated T-cell lymphoma (EATL), one of the most serious malignancies associated with CeD. Given its rarity yet clinical importance, RCD requires discussion as both a diagnostic challenge and a condition with significant oncologic implications.

## 3. Refractory Celiac Disease

RCD is defined by the persistence of symptoms and villous atrophy for 12 months despite strict adherence to a GFD and the exclusion of any other causes of malabsorption [48,49]. According to Ilus et al., RCD affects 0.3% of patients diagnosed with CeD, and its prevalence in the general population is 0.002% [50]. The factors identified as predisposing factors for the development of RCD include older age, malabsorption, seronegativity at the time of CeD diagnosis, and a history of dietary lapses [50,51].

Based on the number of aberrant IELs and clonal rearrangement of the T-cell receptor (TCR), two types of disease are distinguished: RCD type 1 (RCD-1) and RCD type 2 (RCD-2) [50]. Distinguishing between RCD-1 and RCD-2 is crucial, owing to their different treatment strategies and prognoses. The immunological and molecular mechanisms underlying RCD-1 are believed to resemble those seen in uncomplicated CeD. RCD-1 is associated with the normal expression of CD3 and CD8 surface markers on lymphocytes without clonal TCR rearrangement. To date, no somatic genetic alterations have been reported in RCD-1. RCD-1 distinction from CeD is based solely on clinical grounds (persistence of malabsorption and villous atrophy after 12 months of strict GFD) [52]. RCD-2 is characterized by the massive infiltration of the intestinal epithelium by monoclonal lymphocytes, which make up at least 20% of the total IELs, as measured by the flow cytometry of duodenal biopsy specimens [53]. The abnormal IELs in RCD-2 display a dual immunophenotype, showing both T-cell and natural killer (NK) cell characteristics. These atypical IELs are stimulated to expand by interleukin-15 (IL-15), which is overexpressed by enterocytes in patients with CeD. Unlike lymphocytes in healthy individuals, RCD-2 IELs appear developmentally arrested in an early precursor stage. This blocked maturation is thought to result from the IL-15-induced granzyme B-mediated cleavage of *NOTCH1*, a key regulator of T-cell differentiation [54,55]. Phenotypically, RCD-II IELs express cytoplasmic CD3 and the NK receptor NKp46 but lack surface markers typically found on mature T-cells, such as CD3, CD5, CD4, CD8, or a fully functional TCR [54,55]. Although RCD-2 IELs lack a functional TCR, they often exhibit monoclonal rearrangements in the *TCRγ* gene, while rearrangements in *TCRδ* and *TCRβ* are usually incomplete or non-functional [56]. Recent studies have revealed various somatic mutations that underpin RCD-2 pathogenesis and progression to EATL. The JAK-STAT signaling pathway emerges as the most frequently altered, with gain-of-function mutations in *JAK1* and *STAT3* identified in the majority of cases [54,55,56,57,58]. These mutations are responsible for hypersensitivity to IL-15, promoting the survival and clonal expansion of aberrant IELs. In patients without *JAK1/STAT3* alterations, loss-of-function mutations in negative regulators of this pathway (*SOCS1* or *SOCS3*) have been described [58]. The NF-κB signaling pathway is another key area of dysregulation with inactivating mutations in *TNFAIP3* and *TNIP3*, particularly evident in purified IEL populations [54,55,56,58]. Additionally, frequent mutations in epigenetic modifiers such as *TET2*, *KMT2D*, and *DDX3X* have been reported, contributing to genomic instability [54,55,56,57,58]. Moreover, aberrations in DNA repair genes including *POT1* and the dysregulation of *TP53* further compound this instability, whereas CD58 mutations may facilitate evasion of the immune surveillance [54,56,58]. Key genetic alterations in RCD-2 are presented in Table 3.

In a recently published study, Dieckman et al. aimed to investigate the immune environment of RCD-2 beyond the known ILEs. With the use of single-cell mass cytometry, single-cell RNA sequencing, immunofluorescence, and flow cytometry, the authors analyzed duodenal biopsy specimens from RCD-2 patients and controls. They discovered a significant increase in the number of activated memory CD8^+^ T cells (CD27^+^PD-1^+^) and CD4^+^ regulatory T cells in the lamina propria of RCD-2 patients. The CD8^+^ T cells expressed both tissue residency and cytotoxicity markers (CD69, NKG7, PRF1) but lacked CD103, suggesting that they were not intraepithelial. Importantly, these T cells also expressed the inhibitory receptor NKG2A, while the aberrant IELs expressed its ligand, HLA-E, indicating a suppressive interaction that may hinder effective immune clearance of the aberrant clone. The regulatory T cells showed high expression of costimulatory and regulatory molecules (IL1R2, IL32, ICOS, TNFRSF18), further contributing to an immunosuppressive microenvironment [59]. These findings suggest that the balance between immune activation and regulation in RCD-2 is disrupted, potentially allowing clonal IELs to evade immune surveillance. Recent insights into the pathogenesis of RCD-2 highlight its nature as a pre- lymphoma and low-grade lymphoma, with heterogenous molecular alterations and distinctive immune microenvironment driving disease progression to EATL and resistance to conventional therapy [60,61]. In a study conducted by Malamut et al., 33% of patients with RCD-2 developed EATL within 5 years of diagnosis [62]. This rate was even higher in a study by Al-Toma et al., in which 52% of patients with RCD-2 developed EATL within 4–6 years of RCD-2 onset [63]. RCD-2 is also non-responsive to available treatments, and its management relies on a combination of strict adherence to GFD and immunosuppressive therapies. Treatment options for RCD-2 include thiopurines, such as azathioprine or mercaptopurine combined with prednisolone, budesonide, an anti-CD-52 monoclonal antibody called alemtuzumab or cladribine, and autologous hematopoietic stem cell transplantation [52,64]. However, as our understanding of RCD-2 pathogenesis grows, new therapeutic options are emerging. The frequent gain-of-function mutations in the JAK-STAT pathway support the potential use of JAK inhibitors. Furthermore, the expression of immune checkpoint molecules such as NKG2A on CD8^+^ T cells—alongside its ligand on aberrant IELs—suggests that immune checkpoint blockade, including anti-NKG2A agents, may be effective. Future research should focus on assessing the efficacy and safety of these novel therapies, ideally in combination with genomic and immunophenotyping profiling to support patient-tailored approach.

Given that RCD-2 often precedes the development of EATL, further studies aimed at identifying high-risk patients are essential. According to the latest guidelines of the European Society of Gastrointestinal Endoscopy, small-bowel capsule endoscopy, followed by device-assisted enteroscopy, should be performed in cases of non-responsive CeD (persistent or recurrent symptoms despite following a GFD for 6–12 months) or in RCD after excluding gluten ingestion. Device-assisted enteroscopy techniques, including double-balloon enteroscopy (DBE), single-balloon enteroscopy (SBE), and spiral enteroscopy (SE), may be both diagnostic and therapeutic [65]. The recently published Guidelines for Best Practices in Monitoring Established Coeliac Disease in Adult Patients also suggest that those suffering from RCD should be closely monitored with repeated duodenal biopsy to assess the RCD type, as well as with repeated small-bowel capsule endoscopy, to rule out complications, especially EATL. However, surveillance intervals remain unclear [48]. A comparison of RDC-1, RCD-2, and EATL is presented in Table 4.

Importantly, while EATL is the most specific lymphoma subtype associated with CeD, patients with CeD also have an increased risk of developing other lymphoproliferative malignancies.

## 4. Lymphomas

Lymphomas are among the most prevalent neoplasms in patients with CeD [66]. As documented in a study by Smedby et al., apart from EATL, CeD is also associated with the risk of non-intestinal T-cell lymphomas with a standardized incidence ratio (SIR) of 3.6 or B-cell lymphomas with an SIR of 2.2 [20,26,27,29,31,67]. Although the association between CeD and lymphoproliferative malignancies is well-established, the estimated risk of lymphoma in patients with CeD varies across studies. In a recently published population-based cohort study from Sweden, the hazard ratio (HR) for lymphoproliferative cancers was reported to be 2.2 during the first year of follow-up and 1.75 after 1 year after the diagnosis of CeD [12]. Similar figures were published in another Swedish study, in which patients with biopsy-verified CeD (villous atrophy, Marsh stage 3) and those with duodenal inflammation with Marsh stages 1–2 showed an increased risk of lymphoproliferative malignancy, with HRs of 2.82 and 1.81, respectively. Interestingly, this study reported that the increased risk did not apply to individuals with latent CeD, defined as those with positive CeD serology and normal mucosa. In this particular CeD group, the risk profile for developing lymphoproliferative malignancies did not differ from that of the general population [23]. Additionally, Gao et al. proved that individuals with CeD had a 5.35-fold increased non-Hodgkin lymphoma (NHL) risk [28]. The highest risk for the development of T-cell lymphoma was observed in males between the ages of 50 and 80, particularly when CeD was diagnosed at the age of 50, highlighting the significance of age and sex as contributing factors [32].

Almost all available studies have reported a poorer prognosis among patients with CeD affected by lymphomas compared to lymphoma patients without CeD. Koskinen at el. reported that patients suffering from CeD had an increased risk of death from lymphoproliferative diseases with an HR of 2.36 after a mean follow-up of 7.7 years [33]. Abdul Sultan et al., who analyzed mortality among patients with CeD, concluded that compared to non-CeD patients, these individuals showed a 0.15% increased risk of death from NHL within 10 years of diagnosis [30]. In addition, patients with T-cell lymphoma have poorer prognoses than those with B-cell lymphoma [68].

### EATL

EATL is a rare but severe complication of CeD that is often preceded by low-grade clonal intraepithelial lymphoproliferation in RCD type 2 [32,69]. Its annual incidence is approximately 0.4 to 1.0 cases per 100,000 person-years in the general population [70]. The median age at presentation is over 60 years, with a male predominance [55]. According to the World Health Organization Classification of Tumors of Hematopoietic and Lymphoid Tissues, two types of EATL can be distinguished, and type one is strongly associated with CeD [71]. The identified risk factors for EATL include *HLA-DQ2* homozygosity, which was reported in 53.3% of patients with EATL and the rs7259292 single nucleotide polymorphism of *MYO9B* [63,72,73]. Moreover, recent studies have indicated that the genetic profile of EATL overlaps significantly with that of RCD-2. EATL is marked by recurrent activating mutations in the JAK/STAT signaling pathway—most commonly affecting *JAK1* (particularly at the p.G1097 hotspot) and *STAT3*—as well as loss-of-function mutations in negative regulators of the NF-kB pathway (*TNFAIP3* and *TNIP3*) [74,75]. Additional mutations involve genes associated with epigenetic regulation and gene expression (*KMT2D*, *BCOR*, *DDX3X*), mitogen-activated protein kinase signaling (*KRAS*, *NRAS*, *BRAF*), and tumor suppression (*TP53*) [55,76,77].

If diagnosed at the same time as CeD, CeD-associated EATL can be clinically classified as primary, whereas when it is discovered in patients with a history of CeD or RCD-2, it is secondary. The typical clinical manifestations of EATL include abdominal pain, diarrhea, fatigue, and weight loss [69,78,79]. The most commonly affected site is the small intestine (90% of the lesions), specifically the jejunum. Moreover, tumors may occur at various sites, including extraintestinal locations [69,79]. The frequent complications of EATL include obstruction, perforation, and intestinal hemorrhage. Therefore, 6% cases of EATL are diagnosed during emergency surgery [79,80,81].

Treatment of EATL remains challenging and is currently based on chemotherapeutic drugs, such as cyclophosphamide, doxorubicin, etoposide, and vincristine [82]. Chemotherapy may be preceded by the surgical debulking of tumors, and/or followed by autologous stem cell transplantation (ASCT) [83,84]. In a recently published review, Marchi et al. highlighted that patients with EATL may benefit from BV-CHP therapy combined with ASCT. BV-CHP is an advanced chemotherapeutic regimen combining traditional chemotherapy (cyclophosphamide, doxorubicin, and prednisolone) with targeted therapy using brentuximab vedotin. Brentuximab vedotin is an antibody–drug conjugate that targets CD30+ lymphoma cells [85]. Nevertheless, in 50% of the patients, an aggressive treatment approach is not applicable because of their advanced age and low performance status, which is often caused by long-lasting RCD-2 with malnutrition. Considering the side effects and toxicity of chemotherapy, even fewer patients complete treatment [83]. Recently, promising outcomes have been observed in patients treated with a combination of chemotherapy and immunotherapy using the programmed death-ligand 1 checkpoint inhibitor nivolumab. This approach significantly hinders EATL progression and improves overall survival [82,86].

The impact of strict adherence to a GFD on the prevalence of EATL in patients with CeD remains uncertain. Silano et al. have reported the protective role of a GFD in the development of EATL. Among 1, 757 enrolled patients with CeD, nine developed EATL during the follow-up period (SIR: 6.42). However, the SIR for EATL among those strictly adhering to a GFD was lower (2.8), and it further decreased to 0.22 after excluding individuals diagnosed with EATL within 3 years of CeD diagnosis. This period is associated with a higher risk of lymphomagenesis, owing to recent gluten withdrawal [87]. Similarly, in a retrospective cohort study, Holmes et al. reported a lower incidence of malignancy in patients with CeD adhering to a GFD than in non-adherent patients [88]. Nevertheless, NHL has been observed in some patients following a GFD [27]. This may be attributed to prolonged gluten exposure before diagnosis, with insufficient time with the GFD to reverse the effects of long-term gluten consumption. Additionally, following a strict GFD may be challenging because a small amount of gluten may be present in non-cereal food; thus, it is inadvertently consumed by patients.

Considering the aggressive course of EATL with a cumulative 5-year survival of <20% and poor treatment results from the intrinsic chemoresistance of this rare lymphoma, the development of surveillance programs for the early detection of premalignant lesions in CeD is warranted. Nevertheless, to date, no official recommendations have been provided for cancer screening in patients with CeD. However, recognizing the association between persistent villous atrophy and increased risk of lymphoproliferative malignancy, the American College of Gastroenterology issued guidelines that recommend adherence to strict GFD and follow-up biopsy 2 years after the introduction of the diet [3]. The British Society of Gastroenterology has suggested follow-up duodenal biopsy in patients with non-responsive CeD, defined as a lack of improvement in clinical symptoms or laboratory abnormalities after 6 months of GFD or in case of symptoms recurrence despite strict GFD. However, the interval between surveillance examinations remains unclear [89,90]. Moreover, the European Society for the Study of Coeliac Disease has recommended that EATL be excluded in any patient with CeD with abdominal pain, fever, obstruction, anemia, gastrointestinal bleeding, or unexplained weight loss [64].

## 5. Small Bowel Carcinomas (SBCs)

SBC is a rare neoplasm that accounts for <5% of all gastrointestinal malignancies. Patients with CeD have a higher risk of developing SBC, predominantly adenocarcinoma (SBA) than healthy controls [21,22,91,92,93]. Fifty-four percent of SBA cases diagnosed in patients with CeD are classified as adenocarcinoma of the intestinal type and 15% as the medullary type [94]. Ilus et al. found that the risk of developing small intestinal cancer—defined as adenocarcinomas, carcinoid tumors, and stromal tumors, excluding lymphomas—was more than four times higher in patients with CeD compared to the general population [34]. A meta-analysis conducted by Han et al. included 79,991 patients with CeD and showed a statistically significant association between SBC and CeD. This group of patients had an increased risk of developing SBC in the peridiagnostic period, with an odds ratio (OR) of 17.08, compared to an OR of 4.64 in the postdiagnostic period [35].

Vanoli et al. compared histological findings with immunohistochemistry and molecular features of SBC in patients with CeD and Crohn’s disease. The prevalence of microsatellite instability (MSI), a consequence of defective DNA mismatch repair, was significantly higher in patients with CeD (65%) than in patients with Crohn’s disease who developed SBC (16%) or in individuals with sporadic SBC (16%). Additionally, patients with CeD had higher densities of CD3^+^ and CD8^+^ tumor-infiltrating lymphocytes (TILs) than individuals with Crohn’s disease-associated SBC or sporadic SBC, which was associated with a better prognosis (CD3+ TIL: 23.7% vs. 3.3% vs. 5.5%, respectively; CD8+ TIL: 18.6% vs. 1% vs. 4%, respectively). Notably, the study also reported that patients with CeD who developed SBC did not have RCD, which stands in contrast to the typical pathogenesis of EATL [93]. A recently published study from Italy aimed to assess the epigenetic role of LINE-1 hypomethylation, a hallmark of global DNA deregulation frequently observed in cancer, in the development of CeD-associated small bowel adenocarcinoma (CeD-SBA) and compare it with SBAs arising in Crohn’s disease, sporadic SBAs, and non-neoplastic duodenal mucosa in patients with active or treated CeD. The results demonstrated a significant loss of LINE-1 methylation in CeD-SBAs and in the mucosa of untreated CeD, with methylation levels returning to normal after following a strict GFD. Notably, LINE-1 hypomethylation frequently co-occurred with *MLH1* promoter methylation (34% of CeD-SBAs). Despite hypomethylation, LINE-1 activation (ORF1/ORF2 expression) was absent in all cases (except one), suggesting endogenous control of retrotransposon activity in CeD. What is more, the analysis of gene expression revealed that both CeD-SBAs and active CeD showed the strong upregulation of inflammatory genes, including *IL6*, *IL2*, *IL1*, *IL18*, *IFNA1*, *IFN-γ*, and *IL21*. These findings support the hypothesis that CeD-SBAs likely develop in an environment with ongoing immune activity and that LINE-1 hypomethylation is a reversible response to inflammation, rather than a direct cause of cancer development. Therefore, clinically, LINE-1 hypomethylation may serve as a marker of persistent villous atrophy and ongoing immune activation in CeD, potentially aiding histological assessment in follow-up biopsies [95].

Patients with long-standing CeD, particularly those with a late diagnosis, have a higher risk of developing SBC than the general population. The median age at CeD diagnosis among patients who develop SBC ranges from 49 to 59 years [36,94,96]. Moreover, the reported interval between CeD diagnosis and SBC detection is approximately 17 months. Such a short timeframe does not necessarily mean rapid tumor development but rather suggests the presence of a preexisting subclinical malignancy at the time of CeD diagnosis [94]. The initial symptoms of SBC are nonspecific and include abdominal pain, nausea, vomiting, anemia, and gastrointestinal bleeding. Similarly to EATL, SBC can be complicated by bowel perforation or obstruction [97]. Although SBCs can be present in all segments of the small intestine, the jejunum is the most frequently affected site in patients with CeD [14,93].

Complete surgical resection with lymphadenectomy remains the treatment of choice for localized SBC. The type of resection depends on the location of the primary tumor. Duodenal tumors may require either pancreaticoduodenectomy (Whipple procedure) or segmental duodenal resection. Segmentectomy with lymph node dissection is the preferred procedure for jejunal or ileal tumors. In patients with stage II or III disease, surgical resection should be followed by adjuvant chemotherapy. Observation or 6 months of adjuvant treatment with 5-fluorouracil/leucovorin (5-FU/LV) or capecitabine is recommended for T3, N0, and M0 (stage IIA) tumors that are microsatellite-stable (MSS) or mismatch repair-proficient (pMMR) and have no high-risk features. Observation or 6 months of adjuvant treatment with 5-FU/LV/oxaliplatin (FOLFOX), capecitabine plus oxaliplatin (CAPEOX), 5-FU/LV, or capecitabine is recommended for stage II tumors that are MSS or pMMR and have high-risk features (e.g., T4 stage, close or positive surgical margins, and few lymph nodes examined). Six months of adjuvant treatment with FOLFOX, CAPEOX, 5-FU/LV, or capecitabine is recommended for any locally advanced small bowel adenocarcinoma with positive lymph nodes (stage III) [97]. In stage IV, systemic chemotherapy is the only recommended treatment. The recommended first-line regimens are FOLFOX, CAPEOX, FOLFOXIRI, and FOLFIRINOX, all of which may be combined with bevacizumab. Patients with deficient DNA mismatch repair or high-MSI tumors may be administered nivolumab, with or without ipilimumab [97,98].

SBC is commonly diagnosed in an advanced stage, owing to late-presenting symptoms and diagnostic difficulties, and exhibits a poor prognosis with a median overall survival time of 20 months [99]. However, patients with CeD have higher SBC survival rates than those without CeD; therefore, Caio et al. have suggested that CeD-associated SBC may have a less aggressive course [14]. Nevertheless, since no specific screening program for SBC in CeD has been implemented, physicians should pay close attention not only to high-risk individuals, such as those diagnosed after the age of 40 years with persistent symptoms despite a strict GFD, but also remain vigilant in patients without RCD, as SBC may develop independently of RCD. This contrasts with the typical pathogenesis of EATL and suggests that SBC and EATL develop through distinct mechanisms [14].

## 6. Diagnostic Approach and Future Perspectives on Screening for Intestinal Malignancies in Celiac Disease

Diagnosis of malignant complications of the small intestine in patients with CeD relies on a combination of imaging and endoscopic techniques. In recent years, cross-sectional modalities, such as computed tomography (CT) and magnetic resonance (MR) enterography or enteroclysis and fludeoxyglucose F18 (18F-FDG) PET, have played an increasing role in the evaluation of small bowel malignancies. These methods help to localize lesions, assess their severity, and detect extraintestinal complications. Endoscopic techniques include small-bowel capsule endoscopy (SBCE) and single- and double-balloon enteroscopy (SBE and DBE). The latter two methods provide distinct advantages for assessing superficial mucosal lesions and for biopsy sampling for histological evaluation. However, because endoscopic evaluation is invasive and requires specialized equipment and expertise, it may not always be accessible. Moreover, it does not allow for the assessment of submucosal and extraenteric abnormalities. Hence, noninvasive and accurate imaging methods are increasingly needed to evaluate small bowel malignancies in patients with CeD.

In older patients, CT is generally preferred over MR imaging because of its shorter imaging time. Moreover, it provides higher spatial resolution and less variability in image quality than MR. CT also requires fewer breath-holds; therefore, it is an alternative to MR for the evaluation of acutely ill patients and those who have difficulties holding their breath [100,101]. Mallant et al. have reported that intestinal wall thickening, lymphadenopathy, intussusception, and hyposplenism (<120 cc) on CT scans could indicate the development of RCD-2 or EATL [102]. However, CT has several limitations. Intermittent spasms or peristaltic contractions during CT examination can be misdiagnosed as small bowel lesions [101]. Moreover, patients undergoing CT are exposed to ionizing radiation. Therefore, CT cannot be performed frequently, making it an unsuitable tool for disease monitoring.

CT enterography or enteroclysis and MR enterography or enteroclysis are relatively new imaging techniques. They allow small bowel visualization using contrast agents administered orally (CT/MR enterography) or through a nasojejunal tube (CT/MR enteroclysis). Nevertheless, these techniques have been scarcely studied in patients with CeD complications [103,104,105]. Boudiaf et al. evaluated the use of CT enteroclysis in 107 patients with different small bowel diseases. They reported that among 14 patients with RCD, CT enteroclysis facilitated the detection of disease complications in six individuals (one case of jejunal adenocarcinoma, two cases of lymphoma, and three cases of ulcerative jejunitis) [106]. Van Weyenberg et al. documented the usefulness of MR enteroclysis in identifying patients with RCD-2 who were at risk of developing EATL. The presence of fewer than ten jejunal folds over a 5 cm segment of the small bowel, along with mesenteric fat infiltration and diffuse bowel wall thickening, was indicative of RCD-2. The presence of two of these three features showed a sensitivity of 87% and a specificity of 96% for diagnosing RCD-2 [107]. MR enterography was used in a study that investigated the morphological appearance of histologically confirmed small bowel lymphomas in ten patients, among whom six had underlying CeD diagnosed by biopsy. Celiac-associated NHL tended to localize in a single, long (>10 cm), smooth, continuous bowel segment, often with aneurysmal loop dilatation, without a distinct mesenteric or antimesenteric distribution [108]. Radmard et al. analyzed the use of MR enterography to detect small bowel lesions in adult non-responsive patients with CeD (persistent or recurrent symptoms despite 6 months of GFD). The obtained results were later compared with the endoscopic, histopathological, serological, and genetic features. The reversal of the jejunoileal fold pattern (reduced number of jejunal folds and increased number of ileal folds) was significantly associated with severe endoscopic (OR = 8.38) and pathological results (OR = 7.36). Therefore, fold-pattern reversal on MR enterography in patients with CeD could indirectly predict RCD [109]. Because enteroclysis and enterography techniques are complicated, CeD has not been thoroughly studied. Thus, research comparing the sensitivity and specificity of these modalities is lacking, and the choice of a particular technique remains controversial. Although enterography provides suboptimal small bowel distention, it may be preferred over enteroclysis because it eliminates the additional radiation exposure associated with tube placement and is better tolerated by patients [100,101].

Another cross-sectional imaging technique that has produced promising results in the evaluation of CeD complications is 18F-FDG PET. Hadithi et al. reported that 18F-FDG PET was more sensitive and specific than CT for detecting EATL in patients with RCD (100% vs. 87% and 90% vs. 53%, respectively) [110].

As up to 40% of intestinal lesions are inaccessible by traditional upper gastrointestinal endoscopy, endoscopic techniques, such as SBCE, SBE, and DBE, play an important role in the diagnostic process [111,112,113]. SBCE enables the visualization of the mucosa of the entire small intestine using a wireless video capsule. It can identify complications of CeD, such as EATL or small bowel adenocarcinoma, which commonly occur at the distal site of the small intestine [114,115]. However, SBCE has major limitations, as it does not allow the collection of tissue samples from the intestine or the performance of therapeutic interventions [116]. Capsule endoscopy is contraindicated in patients with suspected bowel obstruction. However, intestinal stenosis can be ruled out by evaluation using cross-sectional imaging techniques performed before endoscopy [100]. A prospective cohort study by Kurien et al. assessed the diagnostic effectiveness of capsule endoscopy in detecting mucosal abnormalities in 69 patients with non-responsive CeD. Based on SBCE, significant findings were obtained in 12% of the patients (two cases of EATL, four cases of RCD, one case of ulcerative jejunitis, and one case of fibroepithelial polyp) [117]. Similarly, Atlas et al. investigated the utility of capsule endoscopy in 42 nonresponsive individuals with CeD and found that the sensitivity and specificity of this procedure for the detection of any degree of macroscopic features of villous atrophy (absence of villi and mucosa scalloping/fissuring/mosaic pattern), as assessed by histology, were 56% and 85%, respectively. In addition, capsule endoscopy allowed the identification of two patients with severe complications of CeD: one case of adenocarcinoma and one case of ulcerative jejunitis without overt lymphoma on histological examination [114]. A meta-analysis of three studies by Elli et al. revealed that the pooled diagnostic yield of push endoscopy and DBE for the diagnosis of small bowel neoplasms and ulcerative jejunitis in patients with complicated CeD was 27% [118]. In other studies, the diagnostic yield of DBE for EATL specifically was between 23 and 24% [119,120]. Recent data have indicated that SBCE and DBE are more effective when performed sequentially; therefore, this strategy should be considered the first-line approach in cases of suspected CeD complications [103]. This could help reduce the number of negative DBE procedures that are longer, more invasive, and incur higher costs than SBCE [121,122].

To date, no sensitive biomarkers for the development of CeD-associated malignancies have been identified. Schiepatti et al. aimed to assess the utility of fecal calprotectin (FC) as a marker for CeD severity and predictor of long-term outcomes. In their retrospective study including 177 patients with uncomplicated and complicated CeD as well as non-celiac enteropathies (NCEs), elevated FC levels (>50 mg/kg) were associated with a lack of clinical and histological response to therapy. Moreover, elevated FC was more frequently observed in complicated CeD and NCEs than in uncomplicated CeD. Although not specific to malignancy, these findings suggest that FC may serve as a non-invasive biomarker of disease activity and indicate potentially more aggressive disease phenotypes, warranting closer clinical monitoring [123]. However, this study has several limitations, and despite its promising results, FC testing in CeD patients is not yet standardized in routine clinical practice. Further prospective multicenter studies are needed to validate its utility, determine optimal cut-off values, and explore its potential role in identifying patients at risk of pre-malignant or malignant complications.

Despite a growing understanding of the pathophysiology of malignancy development in CeD, further research on the genetic and immunohistochemical changes in these cases is needed. Genetic screening can serve as a valuable predictive tool in patients with CeD. For instance, the identification of somatic mutations in the *JAK1–STAT3* pathway may help predict the risk of developing EATL in individuals with RCD-2 [74]. Similarly, data indicating higher numbers of tumor-infiltrating lymphocytes in small bowel adenocarcinoma among patients with CeD could be proposed as a prognostic factor, along with MSI, pending further prospective studies [93]. A recently published study by Schiepatti et al. suggested the valuable diagnostic and prognostic role of flow cytometry in the immunophenotyping of IELs in patients with known or suspected RCD and non-celiac enteropathies. A significantly higher mortality rate was observed among individuals with CeD and aberrant IEL phenotype (adjusted HR 4.2). Moreover, 89% of patients with CeD with an aberrant IEL phenotype died during a median follow-up of 30 months, compared to only 10% of patients with CeD with a normal IEL phenotype. Deaths in the CeD group were attributed to either the development of RCD or progression to EATL [124]. These findings are consistent with those obtained in previous studies, which showed that an aberrant IEL phenotype, detected either by immunohistochemistry or flow cytometry, indicates poor prognosis in patients with RCD. Moreover, despite its higher costs and limited availability, flow cytometry could be a more suitable tool than immunohistochemistry for detecting IELs. It allows differentiation between surface and intracellular CD3 expression, thus facilitating the identification of aberrant IELs [125,126,127,128]. Moreover, a Spanish retrospective single-center study has indicated that the intracellular intensity of CD3 on aberrant intraepithelial lymphocytes is a prognostic factor for progression to overt lymphoma in patients with RCD-2 [129]. Nevertheless, further research should be conducted on the interplay between different genetic mutations and the development of CeD-associated neoplasms. To date, research on the utility of screening for mutations in *the JAK1–STAT3* pathway may be the most promising, as these alterations could serve as potential therapeutic targets for treating RCD-2 and preventing its progression to EATL.

## 7. Other Malignancies Associated with Celiac Disease

### 7.1. Esophageal Cancer

Studies have identified a correlation between squamous cell carcinoma of the esophagus and CeD, with the existing literature primarily addressing their epidemiological association. Swedish studies conducted between 1964 and 1994 and analyzing 11,019 patients with CeD reported that they had a significantly increased risk of esophageal squamous cell carcinoma, with an SIR of 4.2 [21]. Data from Green et al. further support these findings, showing a standardized morbidity ratio of 12 for esophageal cancer in 381 patients with CeD [27]. Another study by Van University Medical Center in Turkey conducted from 2012 to 2016 documented similar outcomes and demonstrated a statistically significant prevalence of CeD in patients with esophageal squamous cell carcinoma [130]. Additionally, a meta-analysis by Han et al. showed that patients with CeD had a higher risk of developing esophageal cancer in the peri-diagnosis rather than the post-diagnosis period of CeD [35]. A case–control study by van Gils et al. identified 349 patients with histologically confirmed CeD among 301,425 individuals with malignant lymphoma or gastrointestinal carcinoma from a nationwide Dutch population-based pathology database. The study showed an increased risk of esophageal squamous cell carcinoma, with a relative risk of 5.9 which was restricted to women, particularly those diagnosed with CeD over the age of 50 years [32]. Despite these epidemiological associations, molecular studies investigating how CeD may contribute to the development of esophageal squamous cell carcinoma are notably lacking, highlighting the need for further research in this area.

### 7.2. Colorectal Cancer

The relationship between CeD and colorectal cancer has been examined in several recent studies, with contradictory results. A recently published retrospective population-based study showed that CeD is associated with an increased risk of colorectal cancer. Onwuzo et al. analyzed 47,400,960 individuals with and without colorectal cancer using electronic health records from twenty-six major integrated healthcare systems in the United States and found that 84,360 patients had CeD. Using univariate analysis, researchers calculated that the OR for developing colorectal cancer in patients with CeD was 14.02 [16]. Similar outcomes were reported by Ilus et al., who examined 32,439 individuals with CeD for malignancies. The standardized incidence ratio for colon cancer was 1.35 [34] Goldacre et al. also identified a positive association between CeD and colorectal cancer with an OR of 1.16 [24]. An even higher OR (2.95) was reported in a retrospective case–control study by Lasla et al. [37]. However, a meta-analysis by Han et al., which included 17 studies, did not identify a significant association between CeD and colorectal cancer [35]. Contrasting findings have also been reported by Volta et al. In a multicenter study involving 1757 patients with CeD, these individuals had a lower risk of colon cancer than the general population (SIR = 0.29) [39]. These results align with those obtained in other studies, which showed that the risk of colorectal cancer in patients with CeD was not significantly higher than that in the general population [38,131,132]. These inconsistent epidemiological results may stem from differences in study design, the duration of follow-up, and dietary compliance. Importantly, there is also a notable lack of data regarding the age at colorectal cancer diagnosis and overall survival outcomes specifically in patients with CeD. These limitations in current evidence hinder our ability to draw definitive conclusions regarding risk stratification and prognosis. Therefore, further research is needed in well-characterized cohorts with long-term follow-up, incorporating both epidemiological and molecular data, to better elucidate the relationship between CeD and colorectal cancer development.

### 7.3. Pancreatic Carcinomas

Data regarding the association between CeD and the occurrence of pancreatic carcinoma are contradictory. Currently available studies reveal inconsistent findings both in terms of overall cancer risk and its persistence over time. A recent study by Krishnan et al. demonstrated an increased risk of pancreatic cancer in patients with CeD compared to the general population, with this elevated risk persisting beyond the first year following CeD diagnosis [133]. Similarly, a systematic review conducted by Gromny and Neubauer indicated that patients with CeD may have a 1.46 times higher risk of developing pancreatic cancer than the general population. This increased risk was particularly notable in patients with CeD and other malignancies [40]. These findings are consistent with previous data obtained from Sweden and the United States [21,41]. In contrast, a large population study by Elfström et al. found a positive association between CeD and pancreatic cancer during the first year of follow-up among patients with biopsy-confirmed CeD, inflammation, or latent CeD (normal mucosa but positive TGA IgA, EMA IgA, or tissue transglutaminase test at the time of biopsy). Each examined patient underwent a small intestinal biopsy and was matched by age, sex, county, and calendar year with up to five reference individuals from the Total Population Register. In the first year of follow-up, the HR for CeD, inflammation, and latent CeD was 10.7 (95% confidence interval [CI], 5.77–19.7), 15.7 (95% CI, 9.31–26.6) and 18.6 (95% CI, 6.17–55.9), respectively. However, this association was no longer significant beyond the initial year [22]. Additional studies have further challenged the association between CeD and pancreatic carcinoma development. Goldacre et al. reported no increase in the risk of pancreatic cancer in patients with CeD [24]. Ilus et al. even documented a decreased SIR for pancreatic cancer in a cohort of 32,439 adult patients with CeD [34]. Therefore, the existing evidence is inconclusive and further large-scale, prospective studies are needed to clarify the age-specific and long-term cancer risk in CeD population.

### 7.4. Hepatobiliary Carcinomas

Hepatobiliary carcinomas in patients with CeD have not been extensively studied. The previously mentioned research analyzing the Swedish cohort reported HR of 1.80 (95% CI, 1.44–2.25) for hepatobiliary cancer among individuals with CeD, indicating an increased overall risk compared to the general population [12]. Askling et al. documented an increased risk of primary liver cancer (SIR = 2.7), identifying seven hepatocellular carcinomas, two cholangiocarcinomas, and two cases of mixed or other origins among 249 patients with concomitant CeD and malignancy [21]. These findings were later supported by an analysis by Elfström et al., in which the risk of primary liver cancer in patients with CeD was elevated in the first year of follow-up (HR = 6.05) and persisted thereafter (HR = 1.78) [22]. Nevertheless, a deeper insight into this matter is required. Future research should prioritize large-scale, prospective cohort studies to better define the incidence, prevalence, and risk factors of hepatobiliary carcinomas in this population. Additionally, studies investigating the molecular and cellular mechanisms underlying hepatobiliary carcinogenesis in patients with CeD are essential for the development of diagnostic strategies and evidence-based management guidelines.

### 7.5. Thyroid Neoplasms

The data on the risk of thyroid neoplasms in patients with CeD are conflicting. Kent et al. examined 606 patients with CeD and observed a significantly increased risk of papillary thyroid cancer in this group [42]. A positive association between CeD and thyroid cancer was also reported in an Italian study by Volta et al., in which 1757 patients diagnosed with CeD demonstrated a two-and-a-half-fold increased risk of papillary thyroid cancer [43]. Moreover, a higher prevalence of autoimmune thyroid conditions, such as Hashimoto’s disease, was observed among patients with CeD, which may exacerbate the risk of thyroid neoplasms [134]. Meanwhile, two studies from Sweden did not identify a significant correlation between thyroid malignancies and CeD [21,44]. Although the available data on thyroid neoplasms in the course of CeD remain inconclusive, and no studies to date have investigated the potential underlying molecular mechanisms, patients might benefit from undergoing an additional thyroid examination once the diagnosis of CeD is established.

## 8. Conclusions

Most studies have indicated that patients with CeD have a higher risk of developing lymphomas, particularly EATL and small bowel carcinomas, than the general population. However, the findings regarding other malignancies are conflicting and remain focused on epidemiological data. Therefore, despite the increased awareness of the potential complications associated with CeD and the accessibility of more accurate diagnostic tools, our understanding of the risk factors and exact mechanisms leading to the development of certain types of cancers remains limited. The early detection of malignancies in CeD is challenging, owing to nonspecific symptoms and the absence of simple blood, stool, or saliva biomarkers, as well as the fact that regular endoscopic follow-up with biopsies is hardly possible. Moreover, small bowel examination is particularly challenging. Enteroscopy, MR enteroclysis, and MR enterography are not widely accessible and are time-consuming, highly operator-dependent, and burdensome for patients. Therefore, we hope to develop new, highly sensitive, and specific non-invasive diagnostic tools supported by AI. For example, 18F-FDG PET can help distinguish between EATL and RCD-2 with high sensitivity and specificity. Moreover, recent advancements in the understanding of mechanisms underlying oncogenesis in CeD, particularly the development of RCD-2 and its progression to EATL, are opening new diagnostic and therapeutic possibilities. Comprehensive molecular profiling could facilitate early diagnosis by identifying genetic and epigenetic alterations (e.g., *JAK1*, *STAT3*) associated with malignant transformation. Additionally, molecular profiling may support risk stratification, enabling the closer monitoring of high-risk individuals.

**Figure 1:**
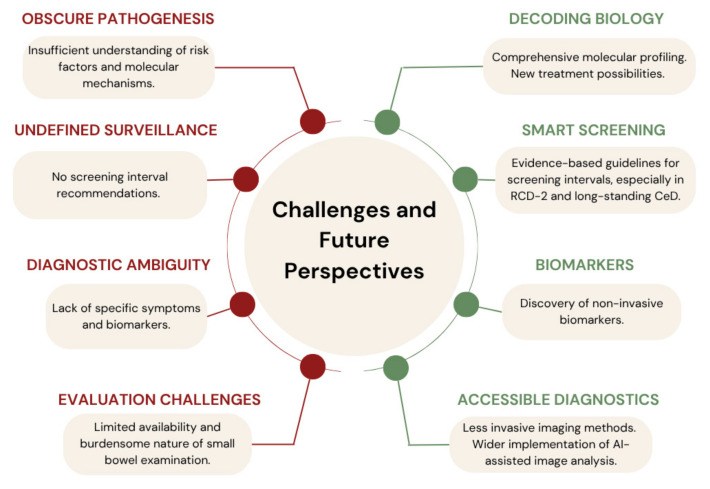
Key diagnostic challenges and future perspectives related to malignancies in celiac disease.

Furthermore, newly identified genetic alterations, which are hypothesized to be the main drivers of CeD-associated lymphomagenesis and small bowel oncogenesis, along with the immunosuppressive tumor microenvironment, represent promising therapeutic targets for blocking cancer progression and restoring immune surveillance. Therefore, targeted approaches using JAK inhibitors and immune checkpoint blockade (anti-PD-1, anti-NKG2A) could be explored as future treatment options, with promising outcomes already documented in EATL, where chemotherapy and nivolumab-based immunotherapy have significantly improved progression and overall survival. Additionally, the upregulation of inflammatory cytokines (e.g., IL-6, IL-1, IFN-γ) in CeD-SBAs supports the role of chronic inflammation as a driver of neoplastic transformation and suggests potential benefits from anti-inflammatory or immune-modulating therapies, particularly in patients with poor dietary adherence or persistent inflammation despite following a GFD. Figure 1 summarizes the main diagnostic challenges and outlines future perspectives in the management of malignancies associated with CeD.

To date, no official recommendations for cancer screening and surveillance have been proposed despite the extremely poor prognosis of malignancies that develop during the course of CeD. Therefore, regardless of the debatable influence of a strict GFD on cancer development, it is currently the only available preventive strategy. Therefore, clinicians and patients should be aware of the importance of adherence to dietary treatment and maintain oncological vigilance in cases of persistent and recurrent symptoms despite 6–12 months of a strict GFD. In addition, as RCD-2 is considered to be a pre-lymphoma state, patients with this condition should be evaluated with extreme caution. Nevertheless, further prospective cohort studies that include molecular profiling are required to assess the risk of malignancy in the peri- and post-diagnostic periods, develop screening strategies, and evaluate the management of patients with CeD and concomitant malignancies, especially EATL or SBC.

## Figures and Tables

**Table 1:** Risk of malignancies and mortality in patients with CeD.

Study	CeD Cases	Risk Factors	Cancer Mortality Rate Among Patients with CeD
Lebwohl et al. [12]	47,241	Diagnosis at age ≥ 60 years: HR = 1.22 (95% CI, 1.16–1.29) Diagnosis between 40 and 59 years: HR = 1.07 (95% CI, 1.01–1.14) Recent CeD diagnosis (first year of follow-up): HR = 2.47 (95% CI, 2.22–2.74)	–
Lebwohl et al. [18]	49,829	–	Increased compared to the general population: HR = 1.29 (95% CI, 1.22–1.36)
West et al. [17]	4732	Recent CeD diagnosis (first year of follow-up): HR = 2.07 (95% CI, 1.45–2.96)	Increased compared to matched controls: HR = 1.31 (95% CI, 1.13–1.51)
Peters et al. [20]	10,032	–	Increased compared to general population: SMR = 1.7 (95% CI, 1.5–2.0)

Abbreviations: CeD—celiac disease; HR—hazard ratio; SMR—standardized mortality ratio; Cl—confidence interval.

**Table 2:** Risk of developing various types of malignances in patients with CeD.

Type of Cancer	Study	Risk/Mortality Assessment	Reference
NHL	Catassi et al.	Increased risk OR = 3.1 (95% CI, 1.3–7.6)	[26]
	Green et al.	Increased risk SMR = 9.1 (95% CI, 4.7–13)	[27]
	Peters et al.	Increased mortality SMR = 11.4 (95% CI, 7.8–16.0)	[20]
	Gao et al.	Increased risk OR = 5.35 (95% CI, 3.56–8.06)	[28]
	Leslie et al.	Increased risk SIR = 6.91 (95% CI, 4.26–8.28)	[29]
	Sultan et al.	0.15% increased risk (95% CI, 0.03–0.27) of dying from NHL from the general population baseline risk	[30]
	Smedby et al.	Increased risk SIR = 6.6 (95% CI, 5.0–8.6)	[31]
HL	Smedby et al.	No increased risk SIR = 1.0 (95% CI, 0.02–5.6)	[31]
T-cell lymphoma	Van Gils et al.	Increased risk RR = 35.8 (95% CI, 27.1–47.4)	[32]
All types of lymphoma	Koskinen et al.	Increased mortality HR = 2.36 (95% CI, 1.65–3.39)	[33]
	Lebwohl et al.	Increased risk HR = 2.20 (95% CI, 1.94–2.49).	[12]
Small bowel carcinoma	Askling et al.	Increased risk SIR = 10 (95% CI, 4.4–20)	[21]
	Elfstrom et al.	Increased risk after the first year of follow-up HR = 2.22 (95% CI, 1.19–4.14) for Marsh 3 HR = 2.49 (95% CI, 1.07–5.79) for Marsh 1–2 HR = 4.67 (95% CI, 0.53–41.4) for latent CeD (only positive serology)	[22]
	Ilus et al.	Increased risk SIR = 4.29 (95% CI, 2.83–6.24)	[34]
	Han et al.	Increased risk OR = 14.41 (95% CI, 5.53–37.60)	[35]
	Silano et al.	Increased risk SIR = 25 (95% CI, 8.5–51.4)	[36]
	Green et al.	Increased risk SMR = 34 (95% CI, 24–42)	[27]
Esophageal squamous cell carcinoma	Askling et al.	Increased risk SIR = 4.2 (95% CI, 1.6–9.2)	[22]
	Han et al.	Increased risk OR = 3.72 (95% CI, 1.90–7.28)	[35]
	van Gils et al.	Increased risk RR = 3.5 (95% CI, 2.1–5.8)	[32]
All types of esophageal cancer	Green et al.	Increased risk SMR = 12 (95% CI, 6.5–21)	[27]
Colorectal cancer	Onwuzo et al.	Increased risk OR = 14,02 (95% CI, 13.40–14.65)	[16]
	Lasa et al.	Increased risk OR = 2.95 (95% CI, 1.36–6.41)	[37]
	Lebwohl et al.	No increased risk OR = 0.75 (95% CI, 0.41–1.34)	[38]
Colon cancer	Ilus et al.	Increased risk SIR = 1.35 (95% CI, 1.13–1.58)	[34]
	Goldacre et al.	No increased risk (excluding cases occurring within the first year of follow-up) Adjusted rate ratio = 1.23 (95% CI, 0.61–2.20)	[24]
	Han et al.	No increased risk OR = 1.15 (95% CI, 0.86–1.56)	[35]
	Volta et al.	Decreased risk SIR = 0.29 (95% CI, 0.07–0.45)	[39]
Rectal cancer	Ilus et al.	No increased risk SIR = 0.82 (95% CI, 0.61–1.07)	[34]
	Goldacre et al.	No increased risk (excluding cases occurring within the first year of follow-up) Adjusted rate ratio = 1.04 (95% CI, 0.28–2.67)	[24]
	Han et al.	No increased risk OR = 0.90 (95% CI, 0.71–1.14)	[35]
Pancreatic cancer	Gromny and Neubauer	Increased risk OR = 1.46 (95% CI, 1.26–1.7)	[40]
	Askling et al.	Borderline increased risk SIR = 1.9 (95% CI, 0.9–3.6)	[21]
	Landgren et al.	Increased risk RR = 2.27 (95% CI, 1.22–4.23)	[41]
	Elfstrom et al.	Increased risk in the first year of follow-up HR = 10.7 (95% CI, 5.77–19.7) Increased risk after the first year of follow-up (not statistically significant) HR = 1.40 (95% CI, 0.97–2.02)	[22]
	Goldacre et al.	No increased risk after the first year of follow-up Adjusted RR = 0.57 (95% CI, 0.07–2.05)	[24]
	Ilus et al.	Decreased risk SIR = 0.73 (95% CI, 0.53–0.97)	[34]
Hepatobiliary carcinoma	Lebwohl et al.	Increased risk HR = 1.80 (95% CI, 1.44–2.25)	[12]
	Askling et al.	Increased risk SIR = 2.7 (95% CI, 1.3–4.7)	[21]
	Elfstrom et al.	Increased risk in the first year of follow-up HR = 6.05 (95% CI 2.96–12.4) Increased risk after the first year of follow-up HR = 1.78 (95% CI, 1.22–2.60)	[22]
Thyroid cancer	Kent et al.	Increased risk SIR = 22.52 (95% CI, 14.90–34.04)	[42]
	Volta et al.	Increased risk (not statistically significant) SIR = 2.55 (95% CI, 0.93–5.55)	[43]
	Ludvigsson et al.	No increased risk HR = 0.6 (95% CI, 0.3–1.3)	[44]
	Askling et al.	No increased risk SIR = 0.6 (95% CI, 0.0–3.3)	[21]
Breast cancer	Ludvigsson et al.	Decreased risk in the first year of follow-up HR = 0.85 (95% CI, 0.72–1.01) Decreased risk after the first year of follow-up HR = 0.82 (95% CI, 0.68–0.99)	[25]
	Askling et al.	Decreased risk SIR = 0.3 (95% CI, 0.1–0.5)	[21]

Abbreviations: CeD—celiac disease; NHL—non-Hodgkin lymphoma; HL—Hodgkin lymphoma; HR—hazard ratio; CI—confidence interval; SMR—standardized morbidity ratio; SIR—standardized incidence ratio; OR—odds ratio; RR—relative risk.

**Table 3:** Genetic alterations in RCD-2.

Category	Gene/Alteration	Clinical/Functional Significance	Study
JAK/STAT Pathway (Activation)	*JAK1, STAT3, JAK3*	Hyperresponsiveness to IL-15 → proliferation and survival of aberrant IELs, risk of neoplastic transformation	[54,55,56,57,58]
JAK/STAT Pathway (Regulation)	*SOCS1, SOCS3*	Loss of negative regulation of the JAK-STAT pathway, seen in patients without JAK1/STAT3 mutations	[58]
NF-κB Pathway	*TNFAIP3/A20, TNIP3*	Loss of inflammatory control; chronic NF-κB activation supporting IEL survival	[54,55,56,58]
Epigenetic Regulators	*TET2, KMT2D, DDX3X*	Gene expression dysregulation, impaired histone methylation, increased plasticity and genomic instability	[54,55,56,57,58]
DNA Repair	*POT1, TP53* (abnormal expression)	Defective repair mechanisms → mutation accumulation and genomic instability	[54,56,58]
Immune Evasion	CD58	Allows IELs to evade immune surveillance	[54]

Abbreviations: IEL—intraepithelial lymphocytes; IL-15—interleukin-15.

**Table 4:** Comparison between RDC-1, RCD-2, and EATL.

Characteristics	Disease Type		
	RCD-1	RCD-2	EATL
Intraepithelial lymphocyte phenotype	IELs mostly normal —loss of normal surface markers CD3 and CD8: either < 50% by immunohistochemistry or <20–25% by flow cytometry	IELs mostly aberrant—loss of normal surface markers CD3 and CD8: either >50% by immunohistochemistry or >20–25% by flow cytometry	Atypical IELs. CD30 expression present in most IELs
T-cell receptor gamma gene rearrangement polymerase chain reaction	Polyclonal	Monoclonal	Monoclonal
Treatment	Corticosteroids (budesonide) Immunosuppression (e.g., azathioprine)	Corticosteroids (budesonide) Immunosuppression (e.g., azathioprine) Chemotherapy (e.g., cladribine) ASCT	Chemotherapy and ASCT
Five-year survival	80–95%	44–58%	11–20%

Abbreviations: RCD-1—refractory celiac disease type 1; RCD-2—refractory celiac disease type 2; EATL—enteropathy-associated T cell lymphoma; CD—cluster of differentiation, ASCT—autologous stem cell transplant.

## Data Availability

Not applicable.

## Abbreviations

The following abbreviations are used in this manuscript:

CeD	Celiac disease
RCD	Refractory celiac disease
EATL	Enteropathy-associated T-cell lymphoma
SBC	Small bowel carcinoma
IEL	Intraepithelial lymphocyte
TGAs	Antibodies against transglutaminase 2
EMAs	Endomysial antibodies
HLA	Human leukocyte antigen
GFD	Gluten-free diet
TCR	T-cell receptor
DBE	Double-balloon enteroscopy
SBE	Single-balloon enteroscopy
SE	Spiral enteroscopy
SIR	Standardized incidence ratio
HR	Hazard ratio
NHL	Non-Hodgkin lymphoma
ASCT	Autologous stem cell transplantation
SBA	Small bowel adenocarcinoma
OR	Odds ratio
MSI	Microsatellite instability
TILs	Tumor-infiltrating lymphocytes
MSS	Microsatellite-stable
pMMR	Mismatch repair proficient
SBCE	Small-bowel capsule endoscopy
CT	Computed tomography
MR	Magnetic resonance
SMR	Standardized mortality ratio
